# *R*eally *e*xasperating *v*iral protein from HIV

**DOI:** 10.7554/eLife.05169

**Published:** 2015-01-09

**Authors:** James R Williamson

**Affiliations:** Department of Integrative Structural and Computational Biology, The Scripps Research Institute, La Jolla, United Statesjrwill@scripps.edu

**Keywords:** HIV, protein-RNA structure, nuclear export, RNA export, electron microscopy, Rev protein, human, viruses

## Abstract

Two new structures shed additional light on the nuclear transport of viral transcripts.

**Related research articles** Jayaraman B, Crosby DC, Homer C, Ribeiro I, Mavor D, Frankel AD. 2014. RNA-directed remodeling of the HIV-1 protein Rev orchestrates assembly of the Rev-Rev response element complex. *eLife* **3**:e04120. doi: 10.7554/eLife.04120

Booth DS, Cheng Y, Frankel AD. 2014. The export receptor Crm1 forms a dimer to promote nuclear export of HIV RNA. *eLife* **3**:e04121. doi: 10.7554/eLife.04121

**Image** A viral protein called Rev (green) helps to transport viral RNA out of a cell nucleus by binding to part of the RNA (red) and also to a nuclear transporter (grey)

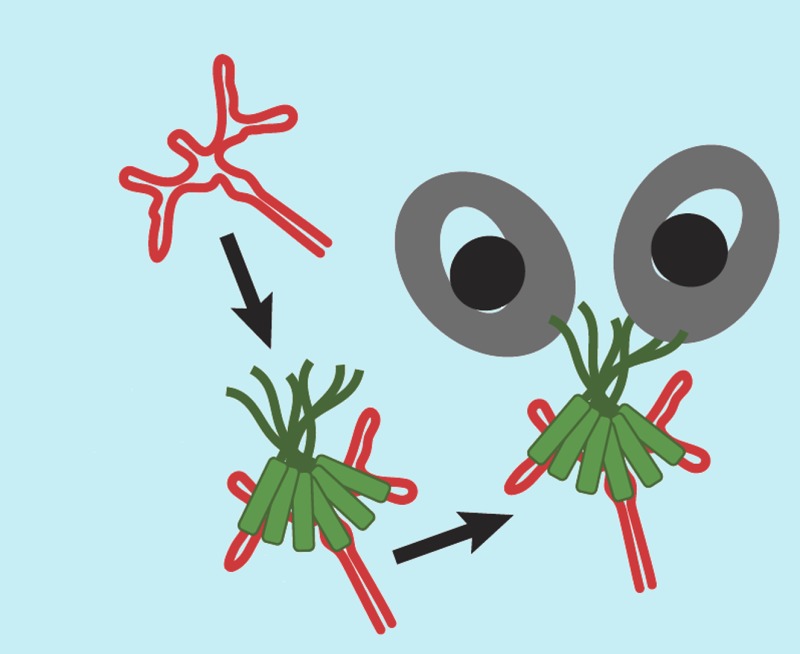

When viruses infect cells, they often use specialized proteins that hijack the host cell's proteins to carry out essential tasks. For example, the human immunodeficiency virus (HIV) needs to transport copies of its RNA out of the nucleus of the host cell and into the cell's cytoplasm in order to create important viral proteins. To do this, a protein in HIV called Rev hijacks a transporter protein that carries cargo across the membrane that surrounds the cell nucleus. As a biochemical entity, Rev has proven to be truly exasperating over the last two miserable decades due to its propensity to generally misbehave—by aggregating or forming fibrils or oligomers—when studied in the laboratory. Now, in *eLife*, two papers by Alan Frankel and colleagues at the University of California, San Francisco provide a more detailed structural picture for how Rev works (Booth et al., 2014; Jayaraman et al., 2014).

Three major regions of the Rev protein are required for it to help HIV replicate: an RNA binding region, an oligomerization region, and a Nuclear Export Signal. The RNA binding region interacts with a part of the viral RNA called the Rev-Responsive Element (RRE). A small (∼30 nucleotide) binding site within this element has a high affinity for Rev, and a larger complex forms from this binding site (Heaphy et al., 1990). The RNA binding region of Rev forms an alpha helix that docks in to a complementary groove in the high affinity binding site on the RRE (Kjems et al., 1990). Adding more Rev proteins onto the RRE results in the formation of a series of larger complexes, but where these additional Rev molecules bind is generally not known. The one exception is a weak secondary Rev binding site that has been identified adjacent to the high affinity site. Truncated RREs are generally less efficient for nuclear transport, and so the consensus is that multiple Rev proteins must bind to the RRE for efficient viral RNA transport.

The oligomerization region of Rev controls the interactions between Rev monomers. Each monomer has two alpha-helical regions that form a hairpin structure, and this structural unit can form complexes with other Rev proteins. The hairpin has two different faces, which were first identified using a genetic screen (Jain and Belasco, 2001). Two previously published crystal structures of Rev dimers have revealed the molecular details of these two interfaces (Daugherty et al., 2010; Dimattia et al., 2010).

The third major region of the Rev protein, the Nuclear Export Signal, is a short peptide motif that interacts with a nuclear transporter called Crm1, thereby indirectly tagging the viral RNA as a cargo destined for export from the nucleus. As Crm1 normally transports human proteins, Rev has effectively hijacked a protein transport pathway to ensure the viral RNA is efficiently transported to the cytoplasm. A number of crystal structures of Crm1 bound to a Nuclear Export Signal peptide have been reported previously (Dong et al., 2009; Guttler et al., 2010), which show that a curved surface of Crm1 mediates this interaction.

The two papers by Frankel and colleagues take significant steps towards understanding how these three functions of Rev—RNA binding, oligomerization, and Crm1 binding—might come together. Yet, significant puzzles remain, and a clear picture of the Rev-RRE-Crm1 complex that exports the viral RNA from the host cell nucleus has yet to emerge.

In the first paper, which features Bhargavi Jayaraman as first author, the first crystal structure of a Rev dimer bound to an RRE RNA fragment is reported (Jayaraman et al., 2014). Many of the interactions seen in this structure fit well within the framework of structures collected by studying smaller parts of the protein individually. Both the high affinity and secondary Rev binding sites on the RRE are occupied as expected, and the architecture of the Rev–Rev dimer interface is clearly seen. In the second paper, for which David Booth is the first author, the structure of a Rev-RRE-Crm1 complex was solved using cryo-electron microscopy, revealing the existence of a Crm1 dimer interface that is unique among reported Crm1-cargo structures (Booth et al., 2014).

Now for the conundrums. First, there are significant differences between the Rev–Rev dimer interface in the Rev-RRE complex reported by Jayaraman et al. and previously determined structures. In particular, the angles between the helices that connect the Rev monomers into dimers are completely different. It is also not entirely clear how the dimer interfaces now observed can be reconciled into a coherent picture of the functional Rev-RRE complex. In principle, the new Rev dimer-RRE structure represents the most advanced molecular model of an intermediate stage of assembly of the final complex. However, the RRE sequence had to be significantly altered to obtain usable crystals for imaging, so we cannot be completely certain that the complexes formed are functional. Furthermore, the data are consistent with the possibility that Rev:RRE could take on multiple different arrangements with respect to Crm1, which complicates the structural analysis.

Unfortunately, it is not possible to unambiguously identify the precise conformation of Rev:RRE in the Rev-RRE-Crm1 complex to help produce a more complete molecular model because large parts of Rev and the RRE are not visible in the structure, presumably due to disorder or because they can take on multiple conformations. It is also not yet possible to understand from the RRE fragments how the Rev dimers in the crystal structure might be arranged in the context of the full RRE. Therefore, the question of the overall architecture of the export complex remains unanswered.

The papers by Jayaraman et al. and Booth et al. represent tremendous advances in a slowly evolving story of how viral transcripts are exported by the Crm1 pathway. The new glimpses of molecular details that have been obtained in these papers will serve to generate the next generation of hypotheses. It may turn out that a Rev dimer or tetramer on the RRE is sufficient to recruit the Crm1 dimer that is the functional export complex. It is important to recall that the Rev concentration in HIV infected cells is very low, and may well be below the concentration necessary to form the menagerie of complexes that have been experimentally generated over the past two decades. Frankel, Jayaraman, Booth and co-workers are to be congratulated on their accomplishments in these most recent studies. However, I personally remain frustrated about how all of these divergent complexes can be understood in a coherent molecular picture. Rev remains for the moment a *R*eally *e*xasperating *v*iral protein.
